# Metastatic Gallbladder Adenocarcinoma to the Endometrium: A Case Report and Review of Literature

**DOI:** 10.7759/cureus.5258

**Published:** 2019-07-28

**Authors:** Justin Oh, Michael Steel, Christopher Conklin, Christina Aquino-Parsons

**Affiliations:** 1 Radiation Oncology, British Columbia Cancer Agency - Vancouver Cancer Centre, Vancouver, CAN; 2 Pathology and Laboratory Medicine, Vancouver General Hospital, Vancouver, CAN; 3 Pathology, British Columbia Cancer Agency - Vancouver Cancer Centre, Vancouver, CAN

**Keywords:** gynecologic oncology, gallbladder malignancy, meta-synchronous metastasis

## Abstract

Gallbladder carcinoma (GBC) metastasis to the uterine cervix is very rare, accounting for less than 10 reported cases. GBC is an uncommon neoplasm with a poor prognosis. Many patients remain asymptomatic until it reaches an advanced stage or discovered incidentally. Most metastatic diseases occur in the lung, liver, and bones. We report a case of a patient treated for GBC with a good clinical response, who presented with metastasis in the uterine cervix. Uterine cervix metastasis from any extragenital primary is rare and poses a radiologic, pathologic, and clinical diagnostic challenge. Here, we review and discuss the published literature on uterine cervix metastasis from extragenital sources. Gynecologic clinicians should be wary of these rare presentations of metastatic disease, as the diagnosis can alter the management.

## Introduction

Gallbladder carcinoma (GBC) is an uncommon malignancy with a poor prognosis. In Canada, there were less than 500 cases in 2013, and the five-year survival for all stages is around 20% [1]. Surgical management provides a curative option, but less than 10% of patients present with a curative disease [2]. In the setting of inoperable advanced disease, first-line therapy is gemcitabine and cisplatin, with median overall survival (OS) of 12 months [3]. Typically, metastatic spreads occur in the liver, lung, bone, brain, and distant lymph nodes, accounting for more than 75% metastatic burden [4-5]. This case study features an unusual presentation of metastatic gallbladder cancer in the uterine cervix.

## Case presentation

A 72-year old female presented to her family physician in February 2017 with severe post-prandial epigastric and lower sternal pain. The diagnosis of cholecystitis was made, and the patient was started on antibiotics, which alleviated her abdominal pain. An abdominal ultrasound in April 2017 showed multiple calculi in the gallbladder, the largest of which measuring 1.9 cm, as well as echogenic sludge bile. In July 2017, she had a laparoscopic cholecystectomy. Intraoperatively, there was significant distension and inflammation of the gallbladder, and there were two large lymph nodes below Hartmann’s pouch in a pericaval position. One of these nodes was biopsied. Post-operative pathology showed a > 2 cm adenocarcinoma of the gallbladder, which arose from a segment of the corpus in continuity with the hepatic bed. A 3 mm margin was obtained. Although there was no identifiable lymphovascular invasion or perineural invasion, pericaval lymph node histopathology was positive for metastasis. Final pathologic staging was pT2 N2.

**Figure 1:**
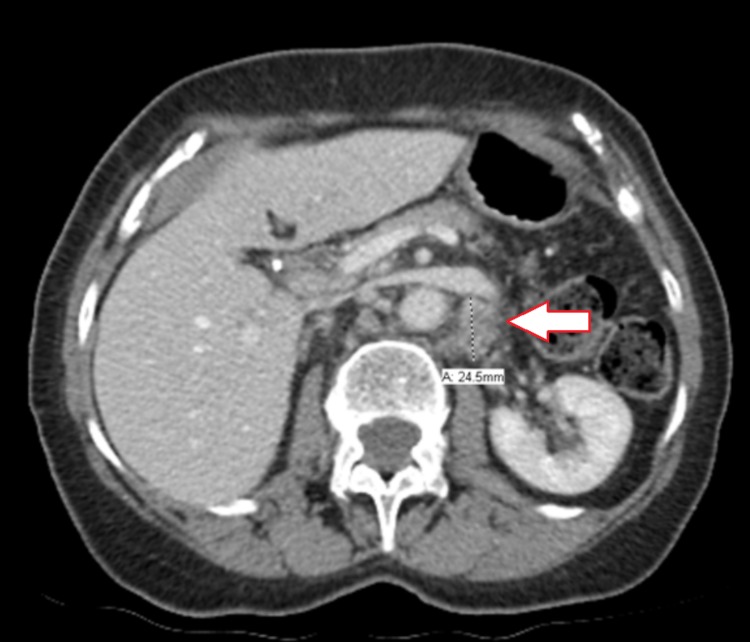
Initial computed tomography (CT) abdomen demonstrating an enlarged left peri-aortic lymph node

**Figure 2:**
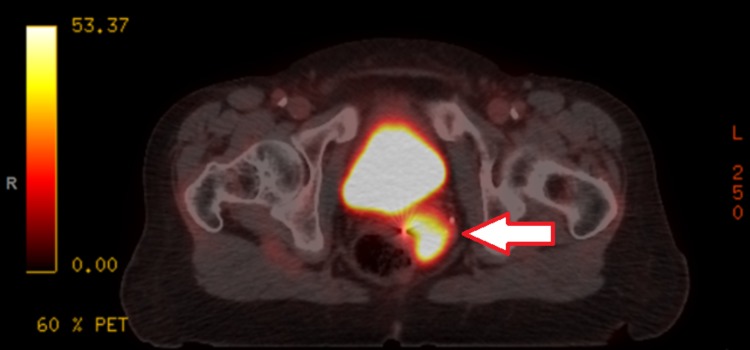
Positron emission tomography (PET) scan demonstrating a fludeoxyglucose (FDG)-avid uterine cervical mass

**Figure 3:**
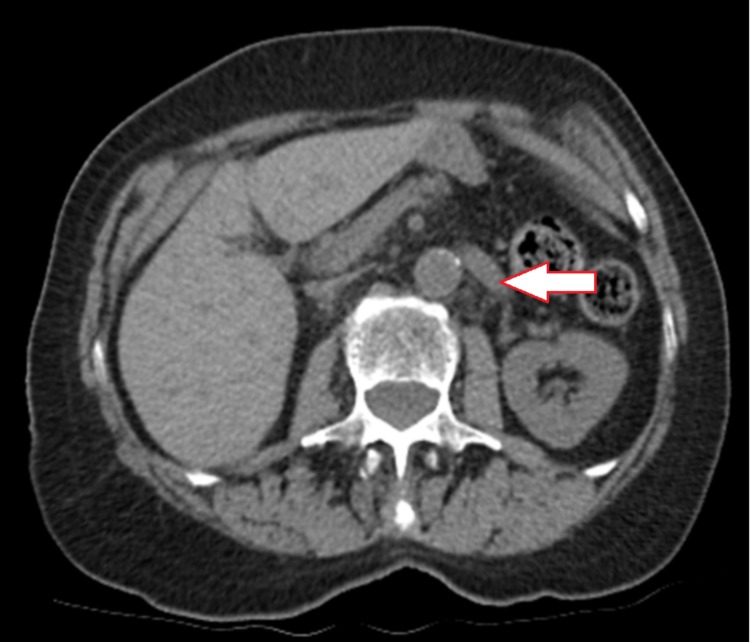
Computed tomography (CT) demonstrating resolution of lymphadenopathies after eight cycles of systemic therapy

A computed tomography (CT) scan of the chest, abdomen, and pelvis in July 2017 (Figure 1) identified multiple mesenteric and retroperitoneal lymph nodes: a right periaortic lymph node measuring 11 x 9 mm and a left mesenteric node measuring 11 x 8 mm. A positron emission tomography (PET) scan in August 2017 (Figure 2) showed multiple fluorodeoxyglucose (FDG) avid para-aortic and aortocaval lymph nodes, the largest measuring 1.3cm with a standardized uptake value (SUV) max of 6.3. There was also a sub-centimeter FDG avid peri-portal lymph node. Uptake in the uterus and adnexal regions were physiologic at that time. The patient was assessed at the provincial pancreas tumor rounds, and the group consensus was for adjuvant systemic therapy. She was started on gemcitabine and cisplatin combination in October 2017 and completed eight cycles in total by March 2018. A CT scan in March 2018 (Figure 3) showed a resolution of the previously observed porta hepatis and aortocaval lymph nodes, with no new lymphadenopathies. Given the promising scan, she elected to continue and eventually completed 13 cycles of chemotherapy.

She was well in follow-up, and a CT scan in January 2019 showed a mildly heterogeneous uterus at the fundus. The pelvic US showed a 2 cm mass in the left side of the uterus and a fluid-filled space on the right side of the uterus measuring at least 2.8 cm x 1.2 cm. During an exam by a local gynecologist, a large and irregular cervix was noted. Background hysteroscopy showed a 1.5 cm fibroid in the left posterior wall. She was referred to a multidisciplinary gynecologic oncology clinic for diagnosis and workup of cervical cancer. She denied any abdominal pain, bloating, or per vaginal bleeding. She had hypothyroidism but was otherwise healthy. She had an unremarkable gynecologic history, was Gravida 1 Para 1, had no history of hormonal replacement, but she did not have regular pap smears. On physical exam, there was an exophytic, vascular mass replacing the entire cervix, measuring 5 cm AP, 5.5 cm LR, and 4cm SI. There was left parametrium involvement, but no sidewall involvement or right-sided parametrium involvement. The pelvis-rectal exam was normal.

**Figure 4:**
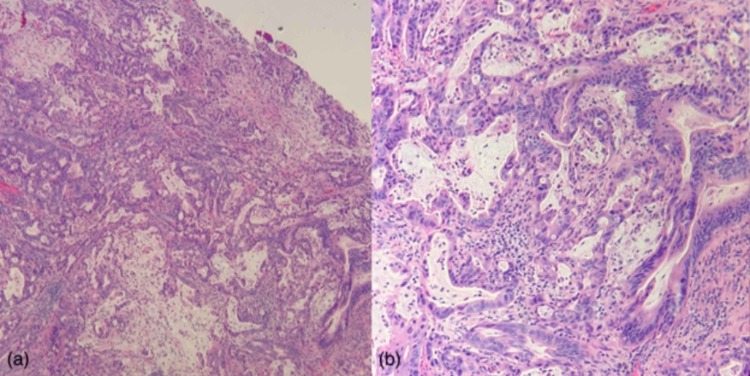
Endometrial/endocervical curetting specimen stained with Hematoxylin and Eosin (H+E), showing a high-grade, infiltrative, mucin-secreting adenocarcinoma at 4X and 10X magnification (‘a’ and ‘b’, respectively). Note the infiltration of the normal endometrial glands by the adenocarcinoma, which produces an associated desmoplastic response. The patient’s previous gallbladder adenocarcinoma showed a remarkable morphologic likeness to that represented herein.

**Figure 5:**
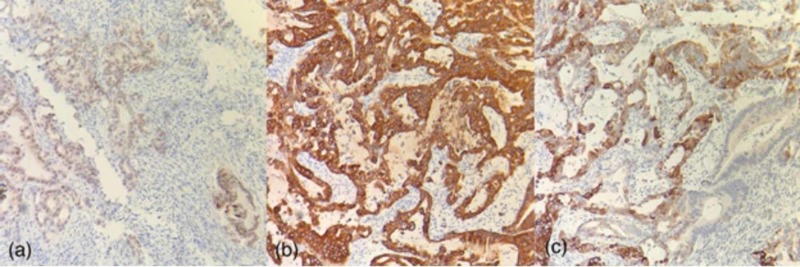
Endometrial/endocervical curetting specimen immunohistochemically stained for caudal type homeobox (CDX2), cytokeratin 7 (CK7), and cytokeratin 20 (CK20) (‘a’, ‘b’, and ‘c’, respectively) at 10X magnification. The cells of the invasive adenocarcinoma are immunopositive for each of these markers, which is in keeping with intestinal-type pancreaticobiliary differentiation; this supports the morphologic impression of gallbladder origin. Note the CK20 immunonegative resident endocervical cells in (c).

**Figure 6:**
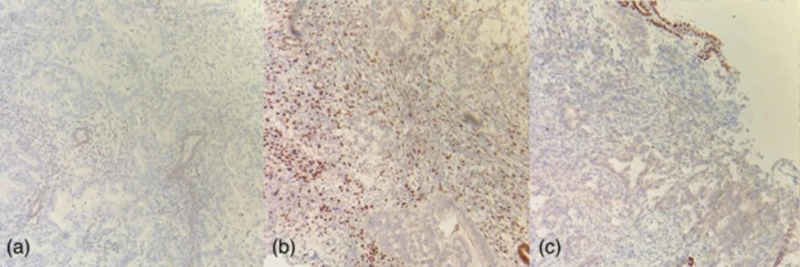
Endometrial/endocervical curetting specimen immunohistochemically stained for estrogen receptor (ER), progesterone receptor (PR), and paired box gene 8 (PAX8) (‘a’, ‘b’, and ‘c’, respectively) at 10X magnification. The cells of the invasive adenocarcinoma are immunonegative for each of these markers, which is inconsistent with Müllerian origin. Note that ER immunochemistry (IHC) stains resident inactive endometrial glands in (a), PR IHC stains endometrial stroma in (b), and PAX8 IHC stains the atrophic surface endometrium in (c), which all serve as positive internal controls. The PR immunonegative gland in (b) represents a displaced, reactive endocervical gland.

A biopsy of the cervix and endometrial curettage were performed. Pathologic review of the biopsy specimens was remarkable for an infiltrating adenocarcinoma with high-grade nuclear features, readily identifiable atypical mitotic figures, mucin production, and a prominent desmoplastic stromal response. While consideration was given to an endometrioid endometrial carcinoma, the histomorphology was remarkably similar to that of the patient’s previous gallbladder adenocarcinoma. The tumor infiltration pattern was also in keeping with metastasis, given the regular intermixing of resident normal/reactive endometrial and endocervical glands (Figure 4) with the tumor. Downstream immunohistochemical workup was performed on the endometrial curetting sample, which showed that the lesional cells were immunopositive for CK7, CK20, and CDX2 (Figure 5) and were immunonegative for PAX8, ER, PR (Figure 6), p16, with a wild type p53 staining pattern. This immunohistochemical profile evidenced intestinal-type pancreaticobiliary differentiation and was supportive of the morphological impression that this endometrial/cervical lesion represented a metastatic adenocarcinoma compatible with the patient's known primary gallbladder adenocarcinoma.

The PET scan in March 2019 showed a new 1 cm, moderately FDG-avid focus in the pancreatic head (SUV max 4.9), a 1.1 cm aortocaval node SUV max of 6.3, and new SUV max 20 uterine mass measuring 8.4 cm. Low-grade FDG uptake in the left external iliac node SUV max 3.4 was also observed. Magnetic resonance imaging (MRI) showed a similar finding of a large uterine mass extending down and involving the entire cervix, with potential involvement of the left parametrium. In the provincial gynecologic tumor rounds, the consensus was that the patient should consider further palliative chemotherapy with gemcitabine and cisplatin.

## Discussion

Gallbladder carcinoma (GBC) is a rare but aggressive tumor that is often found incidentally after a cholecystectomy [6-7]. Otherwise, patients often remain asymptomatic in the early stages. Although incidentally discovered GBC has a better prognosis than symptomatically detected GBC, median survival is still less than 24 months [6]. For locally advanced or metastatic cases, guidelines recommend cisplatin and gemcitabine combination therapy [8]. Although rare, there have been documented cases of complete response (CR) of metastatic gallbladder cancer following cisplatin and gemcitabine combination [3,9]. Our patient had an initial CR of regional lymph nodes to gemcitabine and cisplatin-based on post-treatment CT demonstrating no visible residual disease. When a suspicious mass was detected in the uterus, primary uterine malignancy was suspected until the pathology confirming the metastasis from GBC.

Diagnosis of uterine cervix metastasis from an extragenital primary tumor is rare. Kumar et al. analyzed 63 samples of uterine metastasis from extragenital primary, 43 (68%) of which were autopsy cases [10]. The majority of the cases (>60%) involved the myometrium only, and endometrial involvement was rare. Mazur et al. identified 149 cases of the female genital tract that originated in the extragenital site [11]. The ovary was the most common site of metastasis (76%) followed by the vagina (13%). Combining these two series, breast (33%), colon (20%), and stomach (12%) were the most common primary cancers (Table 1) [10-11]. There were three cases of GBC identified in the series. In total, there are less than 10 cases reporting metastasis of GBC to the uterus or cervix [12].

**Table 1:** Frequency of primary malignancy in the extragenital uterine cervix metastasis in the Kumar and Mazur series

Primary Site	Number of Cases (%)
Breast	25 (33.3%)
Colon	15 (20%)
Stomach	9 (12%)
Pancreas	7 (9.3%)
Lung	3 (4%)
Gall Bladder	3 (4%)
Melanoma	2 (2.7%)
Urinary Bladder	1 (1.3%)
Thyroid	1 (1.3%)
Appendix	1 (1.3%)
Unknown	3 (4%)
Total	75

The mechanism of spread is unclear, but it is suspected that hematogenous is the predominant route of spread in the cases of distant metastasis in comparison to lymphovascular spread when ovaries are involved [13]. Extragenital metastasis to an intragenital site carries a poor prognosis, as it indicates wide dissemination of the malignancy [10]. Patients may or may not have any symptoms from the uterine metastasis, but it is interesting to note that metastasis from colorectal cancer most commonly present with a symptom such as per vaginal bleeding, whereas many of the classically insidious primary cancers, such as pancreatic and stomach, produce no symptoms and could only be detected in autopsies [10].

Our case is unusual in that the patient presented with a large mass spanning the uterus and the cervix as the first recurrence of her GBC, and the exophytic mass on speculum exam was very typical of a primary cervical carcinoma. This case highlights that although rare, clinicians should be wary of the possibility of gallbladder metastases to the uterine body and cervix, as management can be drastically different.

## Conclusions

Gallbladder carcinoma (GBC) is a rare and aggressive disease, which has the tendency to metastasize to distant lymph nodes and the liver. Metastasis to the uterus is an uncommon presentation of malignancy. This case study describes a case of GBC metastasis to the uterine body and cervix. Clinicians should always be wary of uncommon metastatic presentations in patients with a previous diagnosis of malignancy, as management recommendation can change drastically.
